# Molecular Interplay in Cardiac Fibrosis: Exploring the Functions of RUNX2, BMP2, and Notch

**DOI:** 10.31083/j.rcm2510368

**Published:** 2024-10-16

**Authors:** Pavel Docshin, Daniil Panshin, Anna Malashicheva

**Affiliations:** ^1^Laboratory of Regenerative Biomedicine, Institute of Cytology Russian Academy of Science, 194064 St. Petersburg, Russia

**Keywords:** cardiac fibrosis, bone morphogenetic protein 2, Runt-related transcription factor 2, Notch signaling pathways, signaling interactions

## Abstract

Cardiac fibrosis, characterized by the excessive deposition of extracellular matrix proteins, significantly contributes to the morbidity and mortality associated with cardiovascular diseases. This article explores the complex interplay between Runt-related transcription factor 2 (RUNX2), bone morphogenetic protein 2 (BMP2), and Notch signaling pathways in the pathogenesis of cardiac fibrosis. Each of these pathways plays a crucial role in the regulation of cellular functions and interactions that underpin fibrotic processes in the heart. Through a detailed review of current research, we highlight how the crosstalk among RUNX2, BMP2, and Notch not only facilitates our understanding of the fibrotic mechanisms but also points to potential biomolecular targets for intervention. This article delves into the regulatory networks, identifies key molecular mediators, and discusses the implications of these signaling pathways in cardiac structural remodeling. By synthesizing findings from recent studies, we provide insights into the cellular and molecular mechanisms that could guide future research directions, aiming to uncover new therapeutic strategies to manage and treat cardiac fibrosis effectively.

## 1. Overview of Cardiac Fibrosis

Cardiac fibrosis is a pathological process characterized by the excessive deposition of extracellular matrix (ECM) components in the heart, contributing to the structural integrity of the tissue and playing a crucial role in the development and progression of cardiovascular diseases. This condition emerges as a response to various forms of cardiac injury or stress, including myocardial infarction (MI), hypertension, myocarditis, and cardiomyopathies [1, 2, 3].

The process is mediated by cardiac fibroblasts, which, upon activation to myofibroblasts, proliferate and synthesize large quantities of ECM proteins [4, 5]. While fibrosis is initially a compensatory mechanism aimed at maintaining structural integrity, its progression disrupts myocardial architecture, impairs diastolic and systolic function, and predisposes individuals to arrhythmogenesis, thereby contributing significantly to the morbidity and mortality associated with cardiovascular diseases.

Despite its critical role in heart disease progression, current therapeutic options specifically targeting cardiac fibrosis are limited. The complexity of the signaling networks, the diversity of cell types involved, and the dynamic nature of the fibrotic process all present significant hurdles [6]. Therefore, there is a pressing need for novel therapeutic strategies that can target the multifaceted aspects of cardiac fibrosis, offering hope for improved outcomes in patients suffering from this debilitating condition [3].

## 2. Signaling Pathways in Cardiac Fibrosis

Embryonic signaling pathways, such as Notch, transforming growth factor beta (TGFβ) and others, may be involved in the development of cardiac fibrosis as key molecular mediators driving the pathological remodeling of cardiac tissue. The Notch signaling pathway, known for its critical function in cell fate determination and tissue development, may contribute to the complex regulatory network of cardiac fibrosis. For example, neurogenic locus notch homolog protein 3 (Notch3) has been found to negatively regulate the Ras homolog family member A (RhoA)/Rho associated protein kinase (ROCK)/hypoxia-inducible factor 1-alpha (Hif1α) axis, thereby modulating fibroblast activity and potentially attenuating cardiac fibrosis [7]. Several bone morphogenic proteins (BMPs), including BMP2 [8], BMP7, and BMP9, have demonstrated anti-fibrotic effects during cardiac injury [9]. In the context of cardiac fibrosis caused by transaortic constriction, each recombinant BMP has been found to attenuate cardiac fibrosis, mostly BMP7 [10], but the mechanisms underlying BMP’s, anti-fibrotic activity are not fully characterized. This phenomenon highlights a broader range of involvement of embryonic signaling pathways beyond development, highlighting the possibility of their reactivation in response to injury.

Our research has shown that following a MI, there is an upregulation of the Notch signaling pathway and early remodeling factors like BMP2/Runt-related transcription factor 2 (RUNX2) in tissue and cultured cardiac mesenchymal cells [11]. Further experimentation revealed that activating the Notch pathway increased RUNX2 levels [11], although it did not change BMP2 expression [12]. Another study demonstrated that the interplay between BMP2 and Notch pathways varies by cell type and conditions, impacting the synthesis of alpha-smooth muscle actin (α-SMA), a marker for fibrotic transition in cells [13]. These findings suggest that RUNX2/BMP2 and Notch are interconnected in their roles in fibrosis, with their interaction influencing fibrotic changes in specific environments.

## 3. Fundamentals of Notch Signaling in Cellular Differentiation and Development

Several evolutionarily conserved signaling pathways, including Notch, Wingless, Hedgehog, and TGFβ, are instrumental in the development and maintenance of the human body. These pathways are vital molecular mechanisms that operate both independently and in coordination with each other [14]. Notch signaling pathway is critical for a variety of developmental processes, such as maintaining stem cell populations and ensuring tissue homeostasis during the postnatal period.

This pathway is a central regulator of cell fate, influencing the differentiation of multiple cell types including epithelial, neural, endothelial, blood, bone, and muscle cells. Disruptions in Notch signaling can lead to developmental abnormalities and tumorigenesis in humans [15], highlighting the importance of understanding the regulation of this pathway’s intensity and duration.

The highly conserved nature of the Notch signaling pathway is illustrated by the presence of Notch orthologue genes across a diverse range of organisms, including C. elegans, Drosophila Melanogaster, zebrafish, and amphibians. This pathway is composed of several key components: receptors, ligands, target genes, and a transcriptional complex. In mammals, the Notch signaling system comprises four transmembrane receptors (Notch-1, Notch-2, Notch-3, Notch-4), each characterized by three distinct domains: intracellular, transmembrane, and extracellular. Additionally, there are five types of transmembrane ligands involved, including three Delta-like proteins (Dll-1, Dll-3, Dll-4) and two Jagged proteins (Jagged-1, Jagged-2), which interact with these receptors to propagate signaling [16].

Notch signaling involves a complex series of interactions, beginning with the DSL ligand (Delta, Serrate, Lag2) binding to a Notch receptor on a neighboring cell. This binding triggers a two-step proteolytic cleavage of the receptor’s transmembrane domain, catalyzed by the metalloprotease a disintegrin and metalloprotease/TNF-α converting enzyme (ADAM/TACE), which releases the Notch intracellular domain (NICD) into the cytoplasm. Then NICD translocates to the nucleus, where it forms a transcriptional complex with the CSL family transcription factor (CBF1, Suppressor of Hairless, Lag-1) and the Mastermind family proteins, along with coactivator p300, to activate gene transcription [17].

Among its target genes are members of the ‘Hairy and enhancer-of-split’ family, which play a role in inhibiting cell differentiation. A key function of Notch signaling is lateral inhibition, which ensures that neighboring cells adopt different fates to promote diversity in cell types [18, 19]. This is essential in processes such as hematopoiesis, where Notch signaling is crucial for both fetal and postnatal stages [20], and in the differentiation of endothelial cells in synergy with the bone morphogenetic protein pathway [21].

Thus, the Notch signaling pathway is a highly conserved signaling pathway involved in the processes of cell differentiation and development. Its fundamental principles extend their influence on various organ systems, emphasizing the important role of this pathway in a broader physiological context.

## 4. Diverse Roles of Notch Signaling Across Different Organ Systems

The Notch signaling pathway orchestrates a variety of crucial functions in multiple organ systems, demonstrating its broad and essential role in the development and management of disease. In the respiratory system, it guides the differentiation of epithelial cells and development of pulmonary vessels, with disruptions linked to lung diseases such as cancer [22]. It also plays a pivotal role in the central nervous system by maintaining a balance between differentiated and precursor neural cells, which is crucial for neural maturation and diversification [23, 24]

In the renal system, Notch signaling is vital for nephron development during embryogenesis and affects the functionality of mature nephron cells [25]. It governs critical processes in skeletal development by regulating somitogenesis and the differentiation of bone and cartilage [26]. Notch signaling also maintains the epidermis and is integral in the postnatal development of hair follicles, influencing cell differentiation within these structures [27, 28].

Moreover, it plays a role in the sensory development of the inner ear by orchestrating the formation of sensory and supporting cells [29]. In oncology, its impact extends to the regulation of cancer stem cells, highlighting its significance in stem cell biology and cancer [30]. These diverse roles illustrate the critical importance of the Notch signaling pathway in maintaining cellular function and integrity across various biological contexts.

## 5. Implications of Notch Signaling in Cardiac Fibrosis Dynamics

The role of the Notch signaling pathway in cardiac fibrosis presents a complex yet critical area of study, particularly concerning the regulation of fibroblast behavior and fibrotic responses in cardiac tissue. Notch signaling has been observed to mediate diverse responses depending on the cellular context and developmental stage, influencing the progression of cardiac fibrosis.

During neonatal myocardium development, the expression of Notch receptors (Notch1, 3, 4) decreases, a trend that continues into adulthood [31]. In adult hearts, the Notch pathway plays a pivotal role by inhibiting the TGFβ1/mothers against decapentaplegic homolog 3 (SMAD3) signaling axis, thereby reducing fibrotic activity. Specifically, the NICD obstructs the TGFβ1-induced transcription of α-SMA and other ECM components, crucial in the fibroblast to myofibroblast transition [32]. This interaction suggests a dynamic balance between NICD and SMAD3, competing to bind the actin alpha 2 (*ACTA2*) gene promoter, encoding α-SMA, and influencing fibrosis severity [33].

Moreover, experimental models have provided further insights into the pathway’s modulation of fibrosis. *Notch1* was found to modulate the balance between fibrotic and regenerative repair in a mouse model of pressure overload by inhibiting myofibroblast proliferation and enhancing the mobilization and expansion of cardiac muscle precursor cells [34]. This finding, however, is subject to debate; in experiments with Notch transgenic mice experiencing MI and pressure overload, Notch signaling prompted epicardial cells to undergo epithelial-mesenchymal transition (EMT), leading to the formation of a multipotent stromal cell population [35]. These cells could differentiate into fibroblasts and contribute to reparative fibrosis [31].

An *in vivo* study by Boopathy *et al*. [36] demonstrated that delivering the jagged canonical Notch ligand 1 (JAG-1) ligand via intramyocardial injection in rats with MI significantly reduced cardiac fibrosis [37]. Conversely, the suppression of Notch receptors 1, 3, and 4 was associated with an enhanced fibroblast-myofibroblast transition [38]. Additionally, Zhang *et al*. [39] reported that overexpression of the Notch3 receptor, achieved through complementary DNA (cDNA) lentivirus injections, not only improved survival rates in mice but also enhanced cardiac function and reduced the MI-induced increase in cardiac fibrosis.

The regulation of fibroblast proliferation and differentiation by Notch signaling also involves the Notch3 receptor. *In vitro* activation of Notch3 decreases the proliferation of rat cardiac fibroblasts, inhibits their transformation into myofibroblasts, and promotes fibroblast apoptosis. Conversely, knockdown of *Notch3* induces opposite effects. In a rat model of MI, overexpression of *Notch3* mitigates cardiac fibrosis by suppressing the RhoA/ROCK/Hif1α signaling pathway [7].

Further supporting this, knockout studies of the *RBPJ* gene, a critical component of the Notch signaling pathway, in M1 and M2 macrophages have shown a reduction in ECM content and main profibrotic markers (TGFβ1, platelet derived growth factor (PDGF)-B, collagen (COL)1 and COL3) in areas affected by fibrosis [40]. This suggests that modulation of Notch signaling can influence macrophage behavior and fibrotic responses.

The pathway’s effects extend to other aspects of cardiac response. In rat models, Notch activation limits the production of prohypertrophic and profibrotic factors, reducing cardiac hypertrophy and fibrosis following transverse aortic constriction (TAC) [34]. This protective role of Notch highlights its potential in enhancing the heart’s resilience to damage.

A recent study demonstrate that Dlk1, one of the non–canonical Notch ligands, is an important regulator of cardiac pericardium cells. Removal of Dlk1 reduces scar expansion/maturation, whereas overexpression of Dlk1 increases scar expansion/maturation after MI or other cardiac events associated with fibrosis, for example, after heart surgery [41].

**Fig. 1.**
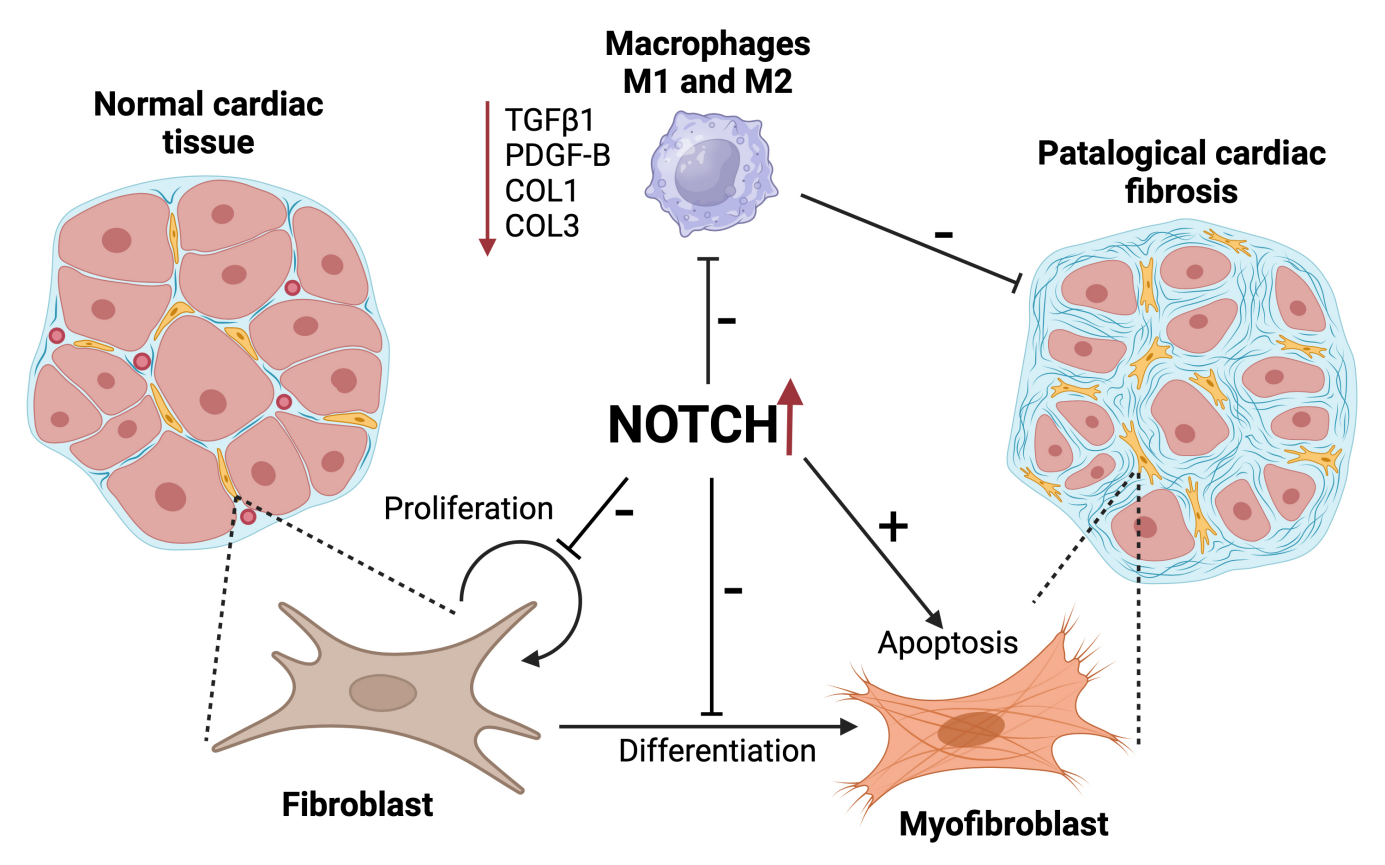
**Visual summary of the role of Notch signaling in cardiac fibrosis**. On the left, normal cardiac tissue is depicted with controlled fibroblast proliferation within the heart tissue, maintaining tissue integrity. In the center, the Notch signaling pathway acts as a critical regulator; upregulated Notch activity is shown to inhibit the secretion of pro-fibrotic markers such as transforming growth factor beta (TGFβ1), platelet derived growth factor B (PDGF-B), collagen 1 (COL1), and collagen 3 (COL3) by macrophages M1 and M2, thus reducing fibrosis. Notch signaling decreases fibroblast proliferation and promotes apoptosis, while inhibiting their differentiation into myofibroblasts, cells that contribute to pathological cardiac fibrosis, shown on the right. The pathological state is characterized by excessive deposition of extracellular matrix components, leading to impaired cardiac function. This figure was created with BioRender.com.

Collectively, these studies underscore the intricate and varied effects of the Notch signaling pathway in cardiac fibrosis, offering insights into potential therapeutic targets for managing fibrotic heart disease (Fig. 1). The pathway’s dual role in inhibiting or promoting fibrosis under different conditions suggests a need for precise therapeutic targeting to harness its beneficial effects while mitigating adverse outcomes.

## 6. BMP2 and TGFβ Dynamics in Fibrosis Modulation

BMP2 belongs to the broader family of proteins known as BMPs, which are part of the TGFβ superfamily [42]. These proteins are critical in various developmental processes and play roles in the pathogenesis of many pathological conditions [43]. Despite their name, which reflects their bone-inducing properties, BMPs are pleiotropic and multifunctional, impacting a wide range of cell types, including those involved in cardiac development and disease [44]. Developmental studies using genetic loss of function models have documented the role of several BMPs in cardiac development and suggested a potential involvement in repair, remodeling, and fibrosis of the infarcted heart, although this area remains underexplored [45, 46].

BMP2 has a well-documented role in the development and formation of bones, inducing differentiation of cardiac cells into cardiomyocytes and stimulating the expression of cardiac-specific genes [42, 47]. Knockout models in mice have shown that *BMP2* is critically involved in cardiac development, particularly in coordinating atrioventricular canal morphogenesis [48, 49]. BMP2 is essential for regulating the EMT of endocardial cells during heart valve formation—a crucial process for proper valvular function and implicated in valvular heart diseases. Research indicates that BMP2, in conjunction with TGFβ2, activates both SMAD-dependent pathways and the Par6/Smurf1 (Smad ubiquitination regulatory factor 1) pathway to facilitate this transformation [50]. These pathways are critical for breaking down tight junctions and enabling the migration and differentiation of cells necessary for valve formation and remodeling.

Beyond skeletal development, BMP2 is crucial in regulating fibrosis across various organs, including the heart, kidneys, and lungs, due to its interactions with TGFβ pathways. In various models, BMP2 has shown potential to reduce fibrosis, often by opposing TGFβ, although there is research suggesting the contrary effect [51]. For instance, in cancer, BMP signaling can enhance TGFβ signaling through the activation of protein arginine methyltransferase 1 (PRMT1). PRMT1 methylates SMAD6/7, which in turn activates SMAD1/3/5, promoting EMT during fibrosis [52].

The study showed a decrease in BMP2 expression in human and experimental fibrotic livers. Notably, adenovirus-mediated delivery of the *BMP2* gene has been effective in reducing liver fibrosis and biliary injury in mice [53]. Mechanistically, BMP2 inhibits the proliferation and migration of hepatic stellate cells by counteracting TGFβ signaling and the EMT. Furthermore, BMP2 has the potential to mitigate TGFβ1-induced renal interstitial fibrosis by reducing Snail expression and reversing the EMT process [54].

Endothelial dysfunction and vascular remodeling contribute to pulmonary hypertension secondary to pulmonary fibrosis by disrupting BMP receptor 2 (BMPR2) signaling, which in turn enhances fibrogenesis [55]. When BMP2 is activated, fibrosis has been shown to be suppressed by reducing the levels of fibronectin, COL1, and COL4 in mouse cavernous endothelial cells under high glucose conditions [56].

In the context of cardiac fibrosis, activated cardiac fibroblasts differentiate into myofibroblasts, key mediators of fibrosis and pathological remodeling [57]. Myofibroblasts, identified by the presence of actin stress fibers containing α-SMA or smooth muscle protein 22 (SM22), form in response to injury or inflammation. Recent research indicates a complex spectrum of fibroblast profiles beyond a simple binary state [58]. Activated cardiac fibroblasts upregulate periostin, a matricellular protein not present in quiescent fibroblasts but robustly expressed after injury. Single-cell sequencing of PDGFRα-expressing fibroblasts post-MI revealed four distinct subpopulations, including proliferative cells and those expressing activation markers like periostin and α-SMA [59]. Additional populations emerge seven days post-infarction, including antifibrotic and profibrotic fibroblasts with ECM-related gene expression [60]. Each fibrotic model exhibits unique temporal and spatial gene expression profiles.

BMP2 counteracts the effects of TGFβ1, a contributor to cardiac fibrosis. In cardiomyocytes, BMP2 promotes the formation of the SMAD6/Smurf1 complex, reversing the pro-fibrotic effects of TGFβ1 by degrading its receptor and inhibiting the TGFβ-dependent activation of SMAD3 and Rho-associated kinase (ROCK) [8]. This complex reverses the pro-fibrotic effects of TGFβ1 by promoting the degradation of its receptor and inhibiting the TGFβ-dependent activation of SMAD3 and ROCK. Additionally, ROCK can suppress BMP2 expression by activating protein kinase C delta (PKCδ).

The role of BMP2 in cardiac fibrosis is further evidenced by its increased expression following myocardial injury. In mouse hearts, BMP2 ligand expression begins to rise within one day after injury, peaks at three days, and then declines [61]. The expression of other BMP ligands such as BMP4, BMP6, and BMP10 is noted to increase seven days post-injury. Additionally, administering recombinant human BMP2 (rhBMP2) or ROCK inhibitors like Y-27632 has been effective in reducing fibrotic markers and improving cardiac function *in vivo* [8].

Furthermore, BMP2 influences myocardial fibroblasts, particularly under stress conditions induced by angiotensin II (Ang II), commonly associated with hypertension and cardiac remodeling [62]. Secreted modular calcium-binding protein 1 (SMOC1) silencing has been shown to suppress fibrosis markers by affecting the BMP2/SMAD signaling pathway in Ang II-treated myocardial fibroblasts, suggesting a protective role against fibrotic signaling in the heart.

**Fig. 2.**
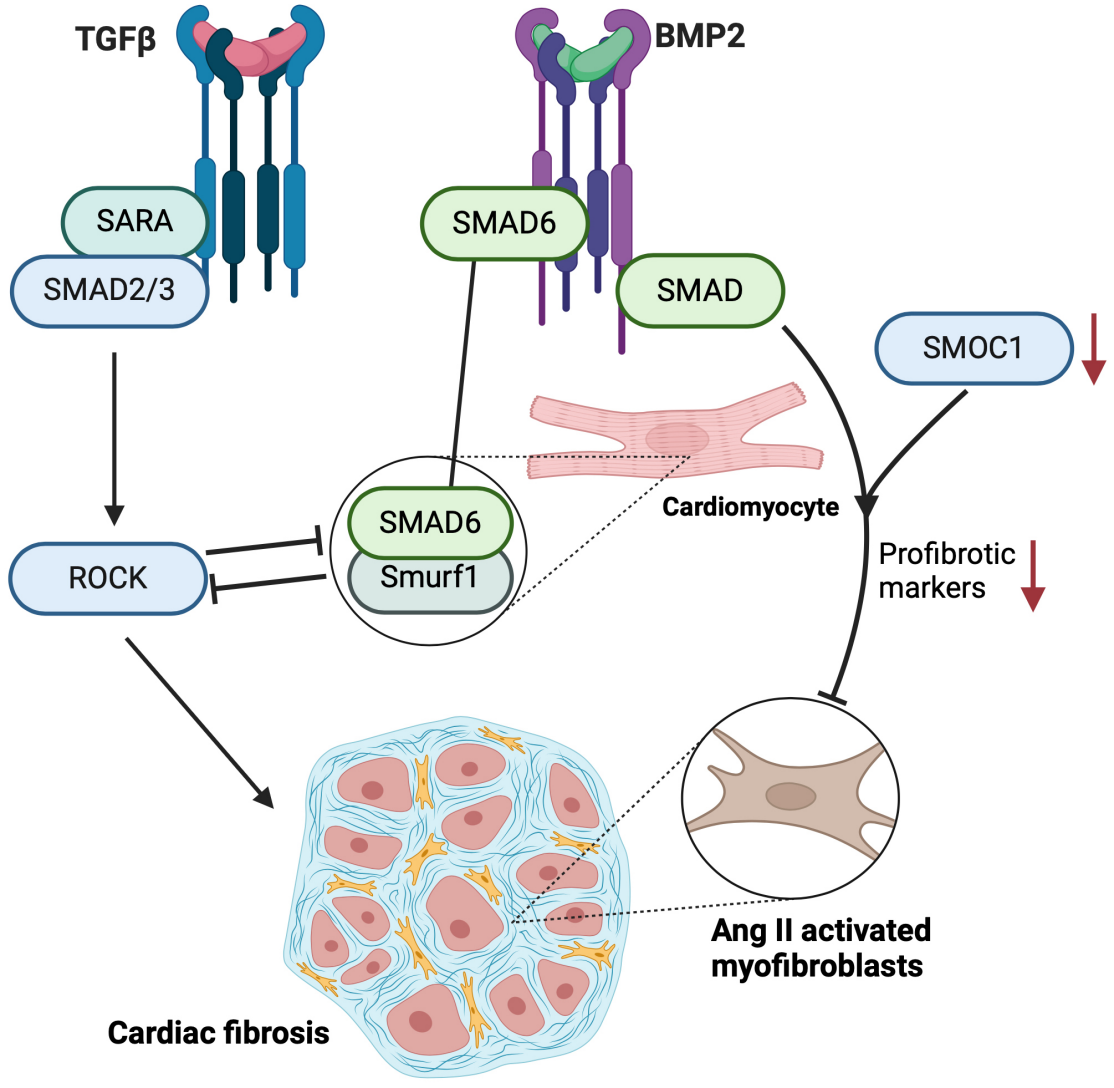
**Schematic diagram of signaling pathways involved in cardiac fibrosis**. It depicts two primary signaling cascades: the transforming growth factor beta (TGFβ) pathway on the left and the bone morphogenetic protein 2 (BMP2) pathway on the right, both converging on SMAD proteins (refers to homologies with C. elegans SMA (“small” worm phenotype) and Drosophila MAD (“mothers against decapentaplegic”) genes), which are critical mediators of fibrosis. In the TGFβ pathway, the receptor is shown activating SMAD2/3 complexed with Smad anchor for receptor activation (SARA). This complex subsequently activates Rho-associated kinase (ROCK), a downstream effector known to promote fibrosis, leading to the pathological remodeling of cardiac tissue, which is illustrated by a cluster of myocardial cells interlaced with fibrotic tissue. The BMP2 pathway involves SMAD6. Notably, SMAD6 forms a complex with Smurf1 (Smad ubiquitination regulatory factor 1), which negatively regulates the TGFβ pathway, providing a potential anti-fibrotic mechanism. Secreted protein acidic and rich in cysteine (SPARC)-related modular calcium binding 1 (SMOC1) is depicted as an inhibitor, shown by a downward red arrow, which suggests that it reduces the activity of SMADs or other intermediaries in the pathway, potentially leading to a decrease in fibrotic response. This figure was created with BioRender.com.

Overall, the interactions of BMP2 with other signaling pathways highlight its complex and multifaceted role in cellular processes and disease states (Fig. 2). These interactions lead to varied outcomes in fibrosis and regeneration, emphasizing the need for continued research to fully understand and potentially harness BMP2’s therapeutic potential in cardiac fibrosis and other fibrotic conditions.

## 7. Multifaceted Role of RUNX2 in Fibrosis and Cardiovascular Regulation

RUNX2 is a key transcription factor integral to osteoblast differentiation and bone formation, orchestrating the gene expression necessary for bone mineralization [63, 64]. Beyond its role in bone health, RUNX2 has broad implications in various fibrotic conditions across different organ systems, demonstrating its versatility beyond skeletal development.

In terms of fibrosis, RUNX2’s involvement is evident in multiple tissue types. In the liver, it activates hepatic stellate cells, which are key players in liver fibrosis [65]. This activation promotes the expression of fibrogenic genes, thus contributing to the progression of conditions such as non-alcoholic fatty liver disease [66]. The use of a selective p38 mitogen-activated protein kinase (MAPK) inhibitor has been shown to ameliorate liver fibrosis through the downregulation of RUNX2 in rat models [67], indicating its pivotal role in fibrogenic processes [68, 69].

In the context of pulmonary fibrosis, RUNX2 expression increases significantly in fibrotic alveolar epithelial type II (AT II) cells, while remaining unchanged in fibroblasts. This differential expression highlights its specific regulatory role in fibrosis-associated gene expression between these cell types [70]. Moreover, RUNX2 deficiency has been observed to exacerbate kidney fibrosis in models of ureteral obstruction by enhancing TGFβ signaling, suggesting a potential protective role of RUNX2 against fibrosis through modulation of this pathway [71].

In diabetes mellitus, RUNX2 contributes to ECM remodeling and aortic stiffening [72]. Specifically, its aberrant upregulation in the aorta can induce medial fibrosis and aortic stiffening through increased expression of matrix proteins like COL1. In rat models of streptozotocin-induced diabetes, fluctuations in blood glucose levels exacerbate aortic fibrosis by affecting the reactive oxygen species (ROS)/p38 MAPK/RUNX2 signaling pathway, highlighting the multifaceted role of RUNX2 in metabolic stress conditions [73].

Shifting to the cardiovascular system, RUNX2 plays a significant role in vascular calcification, where it transforms macrophages into osteoclast-like cells, contributing to abnormal calcification processes [74]. In MI, RUNX2 is significantly upregulated in murine hearts, and is predominantly expressed in myeloid cells, particularly macrophages, at the infarct border zone. This upregulation aids in angiogenesis and vascular repair, illustrating a protective role against adverse cardiac remodeling [75]. Furthermore, RUNX2 activation by Yes-associated protein (YAP) enhances cardiac fibroblast proliferation due to increased ECM stiffness, a critical factor for cardiac remodeling following MI [76]. Moreover, the expression of RUNX2 increases in cardiac fibroblasts cultured on a hard substrate, compared with a soft substrate [76].

Additionally, in the aortic heart valves of pigs and mice subjected to a high-fat and high-cholesterol diet, an increase in RUNX2 expression correlates with elevated ECM production (collagen type i alpha 1 chain (COL1A1)), yet without a corresponding rise in calcification markers. Interestingly, knockdown of RUNX2 prevented excess collagen deposition in aortic valve tissue [77].

**Fig. 3.**
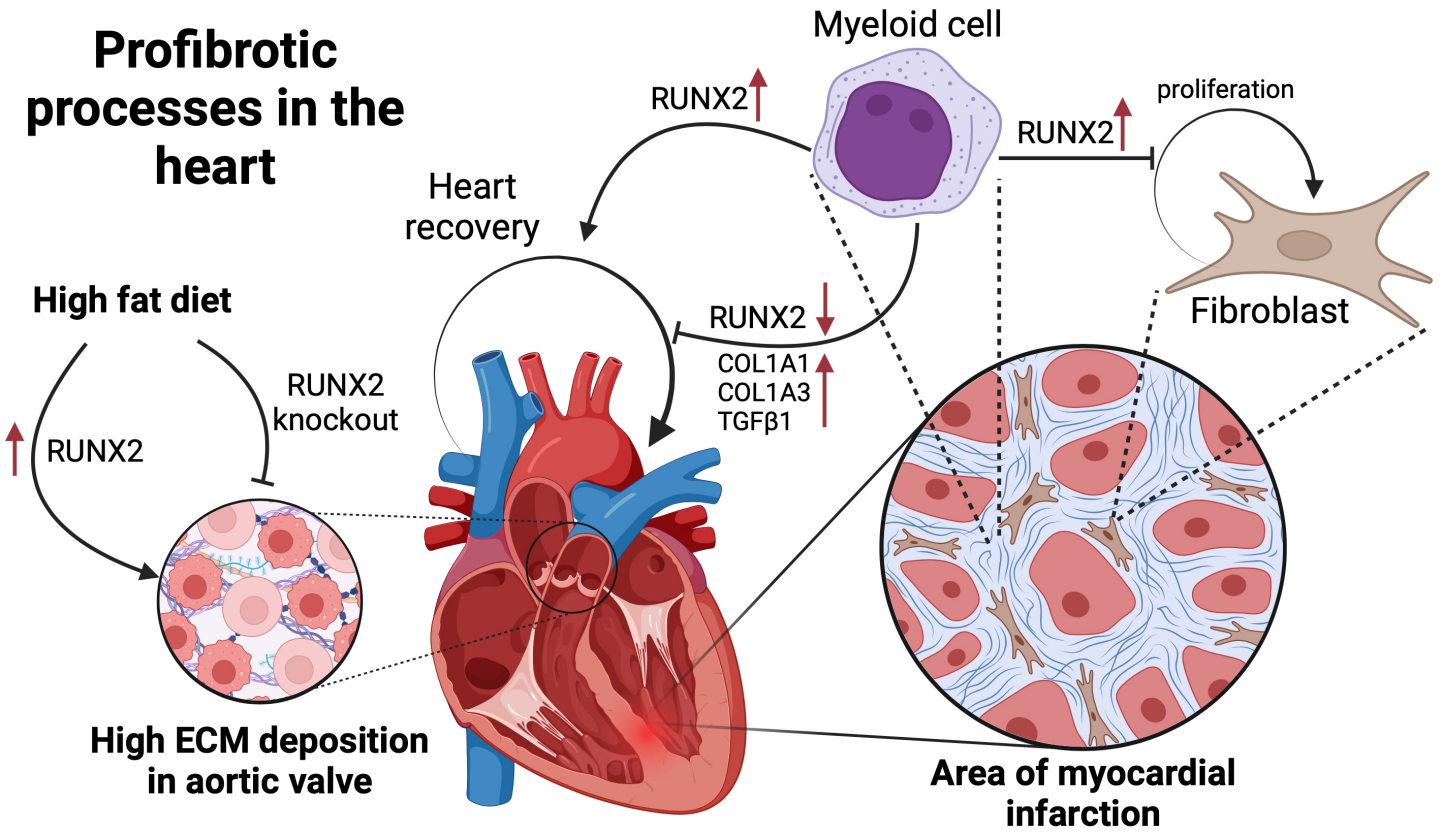
**Visual summary of the role of Runt-related transcription factor 2 (RUNX2) in cardiac fibrotic processes**. It depicts how a high-fat diet can lead to an increase in RUNX2 levels, which in turn results in a high deposition of the extracellular matrix in the aortic valve, symbolizing a profibrotic response. Additionally, the figure outlines the involvement of RUNX2 in myocardial cell repair, where it facilitates the proliferation of fibroblasts following MI. Notably, the diagram presents the dual nature of RUNX2: while its upregulation aids myeloid cell-induced fibroblast proliferation, its knockdown decreases the levels of key fibrotic markers such as alpha-1 type I collagen (COL1A1), alpha 1 chain of type III collagen (COL1A3), and transforming growth factor β1 (TGFβ1). This figure was created with BioRender.com.

These insights collectively underscore the complex and multifaceted role of RUNX2 not only in bone development and mineralization but also in various pathological processes involving fibrosis and calcification across different organ systems (Fig. 3).

## 8. Interplay of Notch, BMP2, and RUNX2 in Cardiac Fibrosis and Cellular Regulation

The interplay between the Notch signaling pathway and BMP2/RUNX2 is central to various biological processes, notably in the context of cardiac fibrosis following MI.

The canonical Notch pathway, through its NICD, counteracts the effects of TGFβ1-induced SMAD3 phosphorylation, reducing the expression of myofibroblast markers and suppressing behaviors of cardiac fibroblasts, such as proliferation and collagen secretion. While TGF-β1/SMAD3 promotes myocardial fibrosis, these processes are moderated by Notch signaling. Techniques like coimmunoprecipitation and immunostaining have demonstrated the molecular interaction between NICD and SMAD3 in cardiac fibroblasts’ nuclei, highlighting the complex regulatory dynamics at play [32].

Within the first 24 hours post-MI, there is an upregulation of Notch signaling components and early remodeling factors such as BMP2 and RUNX2 in response to acute hypoxic stress in both *in vivo* rat models and *in vitro* cell cultures [11]. Activation of the Notch pathway via a lentiviral NICD vector induces a dose-dependent increase in *RUNX2* transcription in cardiac fibroblasts [11, 12]. Additionally, BMP2 may act upstream, influencing Notch target gene *HEY1* (Hes related family BHLH transcription factor with YRPW motif 1) expression in cardiac mesenchymal cells, while NICD enhances BMP2 expression in endothelial cells, promoting endothelial-mesenchymal transition [13].

The interaction between BMP2 and Notch signaling has been studied in various contexts, showing synergistic or antagonistic effects. For instance, BMP2 enhances Notch signaling by increasing the expression of Hes-5 in neural differentiation [78]. BMP2 activates SMAD1, which recruits NICD to form a transcription complex with p300 and CBP-associated factor (PCAF), further upregulating Hes-5, which inhibits proneural bHLH transcription factors like Mash-1 and neurogenin, resulting in the suppression of neurogenesis and promotion of gliogenesis [79].

In the intestinal epithelium, BMP and Notch co-activation guides cell fate decisions, leading to differentiation as cells exit the transit-amplifying zone [80]. In colorectal cancer, BMP signaling remains active in poor-prognosis mesenchymal tumors, where it works with Notch to drive EMT mediated by retained SMAD5 expression [80].

In vascular smooth muscle cells, Notch signaling enhances the effect of BMP2 by increasing the expression of Msx2 [81]. Notch1 intracellular domain boosts BMP2-induced Msx2 expression, with recombination signal binding protein for immunoglobulin kappa J region (RBPJk) and SMAD1 forming a cooperative complex at the Msx2 promoter to regulate osteogenic differentiation and contribute to vascular calcification. Immunohistochemical analysis of human calcifying atherosclerotic plaques reveals colocalized expression of Notch1, BMP2, and Msx2, supporting the *in vitro* findings and suggesting that the cooperative interaction between BMP2 and Notch signaling also occurs *in vivo* [81].

The synergy between Notch signaling and BMP pathways is evident in their cooperative effects on endothelial cell differentiation, which also influences endothelial cell migration through varying degrees of synergy and antagonism [21]. The role of Notch extends further to osteogenic differentiation in human mesenchymal stem cells, where its activation elevates the expression of osteogenic markers like RUNX2, COL1A1, OGN (Osteoglycin), and POSTN (Periostin), reflecting its broad regulatory impact [82].

In contrast, the effects of Notch components such as *HES1* (hairy and enhancer of split-1) and *Hey1/2* (hairy/enhancer-of-split related with YRPW motif protein 1/2) on *RUNX2* activity vary, with *HES1 *enhancing and *Hey1/2* inhibiting *RUNX2* activity [83]. The potential implications of *Notch1* mutations in humans, particularly their effects on *HES1* and *HEY2* expression in the aortic valve and subsequent impact on *RUNX2* activity or the expression of osteogenic genes, offer a promising avenue for further research into the mechanisms underlying calcification of the aortic valve [84].

In osteogenic differentiation, the interplay between Notch and BMP signaling pathways involves complex regulatory mechanisms. Notch activation promotes the degradation of β-catenin and inhibits RUNX2 activity through the actions of HES1, HEY1, and HEYL [85]. Notch inhibits osteogenesis by binding HEY and HES to RUNX2, resulting in an initial promotion of osteogenesis but a subsequent inhibition as differentiation progresses [85]. Meanwhile, BMP signaling activates SMAD1/5/8, MAPK, and c-Jun NH 2-terminal kinase (JNK) pathways, which are crucial for osteogenic differentiation [86]. SMAD proteins form complexes with RUNX2, enhancing its transcriptional specificity and promoting osteogenesis [87]. Additionally, BMP2 can induce the expression of HEY1, which negatively regulates RUNX2, adding another layer of complexity to the regulation of osteogenesis. Notch signaling also directly regulates osteogenesis by upregulating RUNX2, BMP, and JNK. A defect in DLL1/Notch signaling reduces HEY1 expression, affecting BMP pathway activity and osteoblast differentiation [88]. Furthermore, Notch activation is necessary for efficient BMP signaling, as inhibiting Notch reduces Id-1 promoter activity, impairing osteoblast differentiation [89]. These interactions illustrate the intricate balance between Notch and BMP signaling in osteogenic differentiation, where both pathways must be carefully regulated to ensure proper bone formation and development.

In osteogenic differentiation, Notch activation promotes the degradation of β-catenin and inhibits RUNX2 through HES1, HEY1, and HEYL. BMP signaling activates SMAD1/5/8, MAPK, and JNK, resulting in osteogenic differentiation. SMAD proteins form complexes with RUNX2, enhancing transcriptional specificity. Notch inhibits osteogenesis by binding HEY and HES to RUNX2, with early promotion but later inhibition of osteogenesis. BMP2 can induce HEY1 expression, negatively regulating RUNX2. Notch also regulates osteogenesis through direct upregulation of RUNX2, BMP, and JNK. The DLL1/Notch defect decreases HEY1 expression, affecting BMP pathways and osteoblast differentiation. Notch activation is necessary for BMP signaling, as its inhibition reduces Id-1 promoter activity in osteoblast differentiation. Common molecules and targets between these pathways include RUNX2, SMAD proteins, β-catenin, and HEY1, highlighting their collaborative regulation of osteogenesis.

These investigations underscore the intricate crosstalk between Notch, BMP2, and RUNX2 signaling pathways, highlighting their crucial roles in broader developmental and pathological processes. The dynamic interplay between these pathways provides key insights into the cellular mechanisms governing tissue differentiation and disease progression, suggesting potential targets for therapeutic intervention.

## 9. Therapeutic Potential

Cardiac fibrosis, a significant challenge following MI, can potentially be mitigated through targeted therapeutic interventions involving cardiac mesenchymal stem cells (C-MSCs). These cells, also known as cardiac mesenchymal cells, respond dynamically to induction and represent a promising direction for post-infarct heart treatment [11, 90]. For example, cells derived from cardiospheres have demonstrated considerable efficacy in reducing scar tissue and enhancing systolic heart function [91, 92]. Interestingly, the therapeutic potential may not reside in the cells themselves but in their cellular products, such as their secretome [93]. This secretome may interact with embryonic signaling pathways, including Notch and BMP.

Additionally, C-MSCs engineered to overexpress N1ICD (NOTCH1 intracellular domain), when transplanted into the infarcted heart, differentiate into vascular smooth muscle cells. This differentiation leads to significant functional improvement and reduction of fibrosis, surpassing the effects seen with control C-MSCs [94]. Despite low levels of N1ICD expression, CMSC transplantation still exhibits a therapeutic impact [95]. Administering rhBMP2 or ROCK inhibitors like Y-27632 has been effective in reducing fibrotic markers and improving cardiac function *in vivo* [8].

Another critical factor in cardiac fibroblast activation is RUNX2, which may contribute to the exacerbation of cardiac fibrosis [76]. Innovative strategies that target microRNA-129-5p to modulate RUNX2 activity present a novel approach to mitigating the fibrotic effects mediated by this pathway [96]. Such developments underscore the complex interplay of cellular and molecular mechanisms in cardiac fibrosis, highlighting the potential of these emerging therapies to fundamentally alter the progression of fibrotic disease in the heart.

## 10. Discussion

Addressing the contradictory findings surrounding RUNX2, BMP2, and Notch signaling in cardiac fibrosis reveals a complex and sometimes inconsistent picture that is crucial for advancing the field. BMP2 demonstrates significant regenerative potential in specific contexts, such as enhancing cardiomyocyte regeneration in zebrafish, while its inhibition compromises myocardial regeneration [97, 98]. Additionally, BMP2 can reduce markers of fibrosis through activation of PPARβ (peroxisome proliferator-activated receptor beta) and suppression of the profibrotic TGFβ1 [99]. However, BMP2 also exhibits pro-fibrotic effects under certain conditions; for instance, high levels of PRMT1 can enable BMP2 to promote fibrosis by enhancing TGFβ signaling pathways [100]. This dual role of BMP2 highlights the importance of context-dependent signaling in fibrosis, underscoring the need for further research to determine the conditions under which BMP2 acts as a pro- or anti-fibrotic agent.

Similarly, Notch signaling shows conflicting roles in cardiac repair and fibrosis. It supports cardiac repair by enhancing cardiomyocyte proliferation and reducing fibrosis under stress conditions [34]. Conversely, Notch signaling can exacerbate fibrosis by promoting fibroblast-to-myofibroblast transition, and its inhibition is often linked to reduced fibrosis and improved cardiac function post-MI [7]. The interaction between RUNX2 and Notch further complicates the scenario; RUNX2 may inhibit Notch signaling in some cases [101]. Yet, RUNX2 can also work synergistically with Notch to promote fibrosis and osteogenic differentiation in vascular contexts [81]. For instance, RUNX2 activation in hepatic stellate cells has been shown to drive liver fibrosis by upregulating integrin alpha-V (Itgav) expression [66]. Another study highlights RUNX2’s role in promoting aortic fibrosis and stiffness, particularly in the context of type 2 diabetes mellitus [72].

These discrepancies are influenced by several factors, including experimental conditions, cell type-specific effects, and the temporal dynamics of pathway activation. The varying outcomes in different models and cell types underscore the importance of context-dependent signaling. Further research is essential to reconcile these findings and identify the specific conditions under which RUNX2, BMP2, and Notch signaling function as either pro- or anti-fibrotic agents, ultimately guiding more effective therapeutic strategies for cardiac fibrosis.

## 11. Conclusions

The intricate crosstalk among the Notch signaling pathway, BMP2 and RUNX2 plays a pivotal role in the pathogenesis of cardiac fibrosis, influencing the progression and outcomes of cardiovascular diseases. Despite significant progress in understanding the roles of RUNX2, BMP2, and Notch signaling in cardiac fibrosis, several critical gaps remain. For instance, the precise molecular mechanisms through which RUNX2 influences ECM remodeling under different pathological conditions are not fully elucidated. Additionally, the interplay between BMP2 and Notch signaling in various cell types during fibrosis progression requires further exploration.

This review addresses these gaps by integrating recent findings and proposing new hypotheses for future research. Continued research and collaboration will be essential to translate these complex biological insights into effective clinical therapies.

## Abbreviations

ECM, extracellular matrix; EMT, epithelial-mesenchymal transition; C-MSCs, cardiac mesenchymal stem cells; α-SMA, alpha-smooth muscle actin.
